# Timely Reliability Analysis of Virtual Machines Considering Migration and Recovery in an Edge Server

**DOI:** 10.3390/s21010093

**Published:** 2020-12-25

**Authors:** Kangkai Liu, Linhan Guo, Yu Wang, Xianyu Chen

**Affiliations:** School of Reliability and Systems Engineering, Beihang University, Beijing 100191, China; liukangkai@buaa.edu.cn (K.L.); sy1714133@buaa.edu.cn (Y.W.); sy1914203@buaa.edu.cn (X.C.)

**Keywords:** CTMC, end-to-end delay, edge computing, timely reliability, virtual machine

## Abstract

For the edge computing network, whether the end-to-end delay satisfies the delay constraint of the task is critical, especially for delay-sensitive tasks. Virtual machine (VM) migration improves the robustness of the network, whereas it also causes service downtime and increases the end-to-end delay. To study the influence of failure, migration, and recovery of VMs, we define three states for the VMs in an edge server and build a continuous-time Markov chain (CTMC). Then, we develop a matrix-geometric method and a first passage time method to obtain the VMs timely reliability (VTR) and the end-to-end timely reliability (ETR). The numerical results are verified by simulation based on OMNeT++. Results show that VTR is a monotonic function of the migration rate and the number of VMs. However, in some cases, the increase in task VMs (TVMs) may conversely decrease VTR, since more TVMs also brings about more failures in a given time. Moreover, we find that there is a trade-off between TVMs and backup VMs (BVMs) when the total number of VMs is limited. Our findings may shed light on understanding the impact of VM migration on end-to-end delay and designing a more reliable edge computing network for delay-sensitive applications.

## 1. Introduction

With the development of the Internet of Things (IoT), delay-sensitive computing tasks are increasing in cloud computing networks. Because of the long distance between the user equipment and the cloud, dealing with the IoT data in the cloud will cause unacceptable latency, especially for stream or real-time IoT data. To satisfy the strict delay requirements of such IoT services, edge computing emerged. Edge computing provides computing and storage resources at the edge of the network, so that latency-sensitive data and private data can be handled nearby the users, such as applications, e.g., driverless cars, real-time traffic management, virtual reality (VR), augmented reality (AR), and healthcare IoT [1,2]. Regardless of the application scenario, the success of the task relies on the powerful and reliable processing capability of edge computing servers, especially for latency-sensitive applications.

To achieve scalability and robustness, computing and storage resources in cloud servers or edge servers are usually managed by virtualization technology [3,4,5,6]. Computing tasks are performed by independent virtual machines (VMs). Owing to virtualization technology, the network can facilitate load balancing and maintain servers without terminating the task by live migration of VM [7,8]. More importantly, if a VM crashes or is about to crash, the memory image of this VM can be migrated to an idle one and restart the processing, improving fault tolerance of the system. According to [9,10,11], many software failures are transient, which means the system can reboot to repair the problem. If the VM failure is transient, the failed VM can reboot to recover in a short time after the migration. The processing capability of the system is thus recovered. However, as for latency-sensitive tasks, the virtualized management also creates new reliability challenges, which are the failure of VM reducing the processing capability and the migration of VM causing the service downtime [12,13].

Although researchers are trying to design migration mechanisms to shorten the total migration time and downtime, the downtime caused by VM migration still cannot be ignored, especially for service under strict Service Level Agreements (SLAs) [12,14]. As a result, the influence of failure and migration of VM on the satisfying of transmission time requirements needs to be studied, since it is critical for system reliability evaluation, optimizing of resources allocating, and designing of resource management policy. For example, to avoid the server crash, the computing server will not usually assign all the VMs to provide service, but keep a part of VMs idle as backups. When the total number of VMs is limited, more task VMs do not always reduce the delay, since this also brings about more failures in a given time and reduces the backup VMs. Designers of the network and service providers are curious about how to make a trade-off between the task computing resource and idle resource to achieve the highest reliability of the computing network.

The ability of an edge computing network to transmit and process data within the given time can be measured by timely reliability [15,16,17,18]. To analyze whether the VMs in the edge server and the whole network satisfy the time requirements of a task, we define the VM timely reliability (VTR) and the end-to-end timely reliability (ETR), respectively, and propose analysis methods. The end-to-end delay in the computer network consists of four types of delay [19], that is, processing delay, transmission delay, propagation delay, and queueing delay. Since queueing delay is the main random variable of the four components of end-to-end delay [18], we only consider processing delay and queueing delay of the edge server in this paper. From the perspective of edge computing, an edge server can be treated as a multi-server queue with unreliable servers, where the VMs are servers and the tasks are customers. At the same time, to protect the data, the recovery of VMs will not start until the memory image and state have been migrated. To measure the timely reliability of edge computing more precisely, we need to build a queueing model considering failure, migration, and reboot of VM. Present mathematical models [20,21,22,23,24,25,26,27] cannot take the migration of VM into account and neglect the migration downtime. Therefore, to analyze the edge computing system considering migration and recovery of VM, the essential migration-related states of VM need to be defined in a new evaluation model. From the perspective of the network, the end-to-end delay of an edge computing network is determined by the joint distribution of the successive delays of a packet traversing multiple nodes [28]. The main difficulty of networked queueing model analysis is the characterization of the departure process of an upstream queueing node, which is the arrival process of the next queueing node when all intermediate nodes are pure relays [28].

In this paper, to measure the timely reliability of edge computing more precisely, we define three VM states (available, failed-unmigrated, and failed-migrated) and build a multi-server queueing model considering failure, migration, and recovery of VM for edge computing server. The considered failures of VM are all transient and can be recovered by rebooting. The proposed queueing model is analyzed by a continuous-time Markov chain (CTMC) with three types of transfer relations solved by a matrix-geometric approach. The probability density function (PDF) of the sojourn time at an edge computing server is calculated based on the first passage time method. Then, we build a serial queueing model for the edge computing network base on Burke’s theorem, considering edge service and cloud service, respectively. Thereupon, the end-to-end delay and end-to-end timely reliability are obtained. Furthermore, we investigate the impact of VM migration on VTR, the analyzed factors including failure rate, migration rate, reboot rate, and resource allocation. Our results are verified by simulations based on OMNet++.

The main contributions of this paper include: (1) We build a multi-server queueing model that firstly takes account of failure, migration, and recovery of VMs. The present works merely analyze the situation where VMs run perfectly [24,29,30]. (2) A partitioned CTMC method is proposed to analyze three-dimension CTMC and obtain the VM timely reliability. (3) An analysis method based on Burke’s theorem is proposed to obtain the PDF of the end-to-end delay, which is indispensable for the evaluation of end-to-end timely reliability. Present works either obtain average metrics only, such as mean queue length and mean wait time [24,29,31], or obtain the end-to-end timely reliability by simulations [18,32]. (4) We investigate the impact of VM migration and assigning of task VMs and backup VMs on the VTR, which can be a guidance for the designing of a more reliable edge computing network for delay-sensitive applications.

The remainder of this paper is organized as follows. Section 2 presents related works. Section 3 describes the problem, including the analysis of delay at the edge server and the analysis of end-to-end delay. Section 4 proposes the CTMC and end-to-end delay model and proposes an algorithm to obtain the VTR and ETR. Section 5 presents our results, including simulation results and numerical results, and the discussion. Finally, we give the summary and conclusions of this paper in Section 6.

## 2. Related Work

### 2.1. Network Delay Analysis

As mentioned before, the end-to-end delay in the computer network consists of processing delay, transmission delay, propagation delay, and queueing delay [19]. We investigate the literature of the network delay analysis, especially on the queueing delay, and provide a comparative table mainly focusing on the analysis method as below. The main models and methods are described in the table. In Table 1, “Fixed queues” means that the structure of the queue models of a task is fixed. For example, in [24], the queue model is a serial queue consists of the queue of edge computing, the queue of cloud gateway, and the queue of cloud computing; “Stochastic queues” means that the structure of the queue models is not fixed, but determined by some specific mechanisms with probabilities. For example, in [33], tasks might be processed at the IoT layer, fog layer, or cloud layer with different probabilities; “Homogeneous servers” means that the queue servers in the model are all homogeneous, i.e., each server has the same service rate [34]; “Heterogeneous servers” means that the queue servers in the model are heterogeneous [29].

**Table 1:** Comparative table of related works.

Delay	Feature	Description		
Transmission delay	--	Transmission delay is the ratio of the data size and the transmission rate. Factors such as task length, data size, and bandwidth are considered to obtain the transmission delay [31,35,36,37].		
Propagation delay	--	Propagation delay is the ratio of the distance and the propagation speed and is often treated as a function of distance. Factors such as distance [31,33] considered to obtain the propagation delay.		
Processing delay	--	Processing delay depends on the computing capability of the server and the computing complex of the tasks. It is usually modeled by exponential distribution [24,25,29,31,33,35] or constant [18], etc., and acquired by combining with queue delay.		
Queueing delay	Fixed queues	Homogeneous servers	M/M/c	Model the two-stage queues inside a server [25] or multi-stage queues [24,27] of the edge/cloud computing network, consisting of end equipment, edge nodes, and cloud, etc. Queue theory is employed to obtain average metrics such as mean queue length and mean delay, etc. Virtual machines are considered as queue servers [24] and the modeled virtual machine (VM) states only include busy and idle. Server failure of the queue is not considered, and the probability density function (PDF) of end-to-end delay is not obtained.
			Non-M/M/c	Consider tasks arriving with an arbitrary probability distribution [27] or service time following arbitrary probability distribution [30]. Average metrics such as mean queue length and mean delay, are obtained. Virtual machines are considered as queue servers [30] and the modeled VM states only include busy and idle. Server failure of the queue is not considered, and the PDF of end-to-end delay is not obtained.
		Heterogeneous servers	Different resource requests lead to heterogeneous queueing servers. The situation where the task requires VMs with different numbers of cores is analyzed [29,38]. The modeled VM states only include busy and idle. The number of jobs in waiting, the number of jobs under provisioning, the number of busy cores, and the number of jobs in service, etc., are employed to define the state space. CTMC is employed to calculate the mean delay. Server failure of the queue is not considered, and the PDF of end-to-end delay is not obtained.	
	Stochastic queues	Build the queueing models with probabilities according to different offloading mechanisms [31,33]. M/M/c queue model is employed to calculate the mean delay. Server failure of the queue is not considered, and the PDF of end-to-end delay is not obtained.		

As for simulation methods, (1) research focused on the resource management model, such as task offloading and resource allocation, often verifies their delay analysis models by semi-physical simulation [27,33], or Monte Carlo discrete event simulation using tools such as CloudSim [31] and, Arena [29,30]. Average metrics such as mean wait time and mean queue length are calculated to measure the system performance. The influence of VMs state transitions on the timely reliability is not modeled. (2) End-to-end delay models of a network are often verified by Monte Carlo discrete event simulation using Java Modeling Tools (JMS) [24], OPNET [18], OMNeT++, etc. Timely reliability has been analyzed [18,32], but the influence of VM state transitions on the timely reliability is still not modeled.

### 2.2. VMs Timely Reliability Analysis

In the delay analysis of a single node, the main delays include processing delay and queueing delay. Queueing theory is widely used to analyze node delay. Usually, the processes in the computing server are modeled with multiple servers, including homogeneous servers [24,25] and heterogeneous servers [29,39]. An admission manager or load balancer is often modeled as a single server ahead of the computing servers [25,27]. For the task/data arrival, most studies described the task/data flow by Poisson distribution. Researchers then build solutions by constructing and solving a CTMC. For example, Kafhali and Salah [24] modeled the edge server by M/M/s/K queueing model. Pereira et al. [25] proposed a queueing model for a Fog node containing a load balancer and several web servers to solve a closed-form solution. Liu et al. [27] analyzed the user equipment (UE) side delay and server-side delay, respectively, from a perspective of task offloading and resource allocation. However, the above studies only proposed metrics such as utilization of VMs, average throughput, mean response time, average queue length, and discard rate. Measuring the above quantities fails to manifest whether the low-latency constraints of delay-sensitive applications are satisfied. To solve this problem, the PDF of sojourn time at the edge server needs to be obtained.

Moreover, the above analyses based on queueing theory are all under the assumption that the edge node serves perfectly. However, the failure of a server or a VM can reduce the processing capability of the edge node directly and increase latency [12,40,41]. To model the failure and repair of VMs, VMs can be treated as unreliable and repairable queue servers based on the multi-server queueing models with unreliable and repairable servers [20,21,22,23,26]. For instance, Ke et al. [26] and Chakravarthy et al. [23] analyzed multi-server queueing models considering unreliable servers and vacation policy. Although the unreliable and repairable servers in these queueing models are similar to VMs of the edge node, the present multi-server queueing models cannot be applied to model the edge computing servers directly. The reason is that the VM needs to migrate memory images and states before its recovery, resulting in down states for the migration and recovery that cannot be described by present models.

To investigate the influence of VM migration on node delay, some researchers focused on the performance evaluation of VM migration [12,13,40,42,43,44,45,46,47] and latency-aware VM migration algorithms [14,48,49,50,51]. In these studies, migration time and service downtime [13] were measured to evaluate the performance of VM migration, yet how the migration time and service downtime influenced the sojourn time at the edge node has not been studied. For the latency analysis of edge computing, most studies focused on the delay of specific activities, such as processing, task offloading, and resource allocation [52]. The total sojourn time at the edge node has not been well investigated. Liu et al. [53] analyzed the influence of migration, but they only studied the probability that the computing system can provide sufficient VMs. The delay at the server was not analyzed.

All this considered, present studies on VM delay did not investigate the influence of VM migration on the total delay at the VMs, for the queueing models of VMs ignored the failure and migration of VMs [24,25,27], and the VM migration delay was measured separately from the total delay [12,13,14,42,44,45,46,47,53]. Meanwhile, due to the unique process of failure, migration, and recovery of VMs, present queueing models with unreliable servers [20,21,22,23,26] cannot be directly applied to analyze the VM delay. To analyze the timely reliability of the VMs and the network, we build a queueing model considering the whole process of failure, migration, and recovery of VMs and propose a method to obtain the PDF of the delay at the edge server.

### 2.3. End-to-End Timely Reliability Analysis

There are two types of serial queueing models for the analysis of end-to-end delay, which are analytic models and approximate models. By traversal of the end-to-end topology, the end-to-end delay is obtained based on the delay in each node. Some researchers analyzed the situation of two queuing nodes, i.e., tandem queue [54,55,56,57,58]. However, due to the mathematical complexity, the formulation of the networked queueing model is infeasible when the queueing nodes are more than three. Therefore, approximation methods such as simulation [18,59] and the upper bound method [60] are widely applied. Researchers also studied the end-to-end delay of queueing networks based on the departure process approximation. For instance, Xie and Haenggi [28] defined a parameter to measure the spatial correlation between interfacing queueing nodes based on the departure process approximation. Although Kafhali and Salah [24] proposed a serial M/M/s/K queueing model for an edge-cloud computing system based on Burke’s theorem, they did not analyze the end-to-end delay in the model. In addition, Jie Shen et al. [61] proved that the end-to-end delay distribution of a network system is the inverse Laplace transform of the transfer function of the signal flow graph and proposed an end-to-end delay analysis method based on frequency domain. The end-to-end delay analysis considering the structure of the edge computing network is still lacking.

## 3. Problem Description

**Figure 1:**
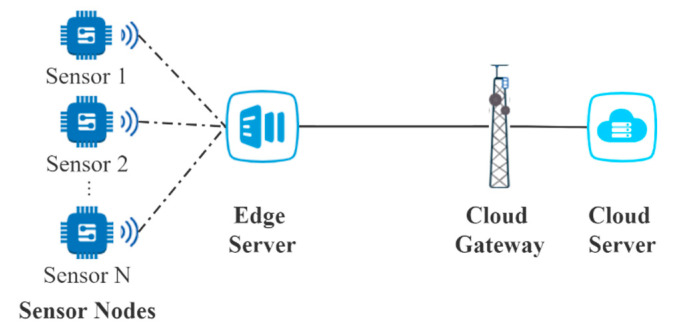
Architecture of the edge computing network.

As depicted by Figure 1, the edge computing network is composed of four parts—sensor nodes, the edge server, the cloud gateway, and the cloud server. The task data generated by sensors are transmitted to the edge server for necessary processing by VMs, including pre-processing, analysis, compression, and encryption, over Bluetooth Low Energy, or Wi-Fi. After the processing at the VMs of the edge server, a part of tasks will be transmitted to the cloud server by the cloud gateway for global storage, power computing, or running various applications. Usually, a task expects to be processed within an extremely short time at the edge server to guarantee the real-time application. Differently, the cloud server can process a task in hours or days. Therefore, the delay at the VMs in the edge server and the delay through the whole network need to be analyzed, respectively. For the edge service, a task only needs to queue at the edge server. Whereas, for the cloud service, a task needs to go through three queues, which are the queue at the edge server, the queue at the cloud gateway, and the queue at the cloud server successively. According to the delay analysis, the definition of timely reliability is given by [18]:

(1) $$R_{T}=\mathrm{Pr}\left(D<D_{max}\right)=\int_{0}^{D_{max}}f\left(t\right)dt$$

where D is the total delay of a task, $D_{max}$ is the maximum allowable delay of the task, f(t) is the PDF of the delay.

### 3.1. Analysis of Delay at the VMs in the Edge Server

In an application, the task data need to be transmitted to the edge server and be processed by VMs within a delay constraint of $D_{max1}$. In the edge server, S VMs are assigned to process tasks, which are called task VMs (TVMs), and d idle VMs are assigned as backups, which are called backup VMs (BVM). All the VMs are homogeneous. Then, the total number of VMs is Z=S+d. The task flow is generated following a Poisson process at each sensor, where the arrival rate is λ. The service time of each VM follows an exponential distribution where the mean service time is 1/μ. If the S VMs are all occupied, the task can be stored in the buffer, whose capacity is K. Then, the maximum capacity of the edge server accommodating tasks is Y=S+d+K. If the buffer is full as well, a discard occurs. We assume that the discarded tasks all violate the delay constraints and do not consider the retrial queue.

**Figure 2:**
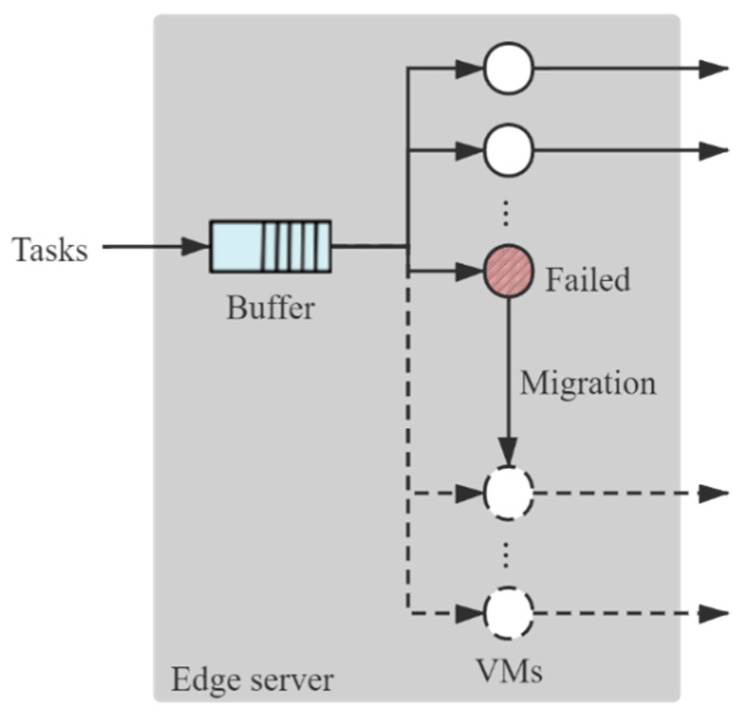
Queueing model of an edge server. The dash-lined circles indicate backup VMs.

**Figure 3:**
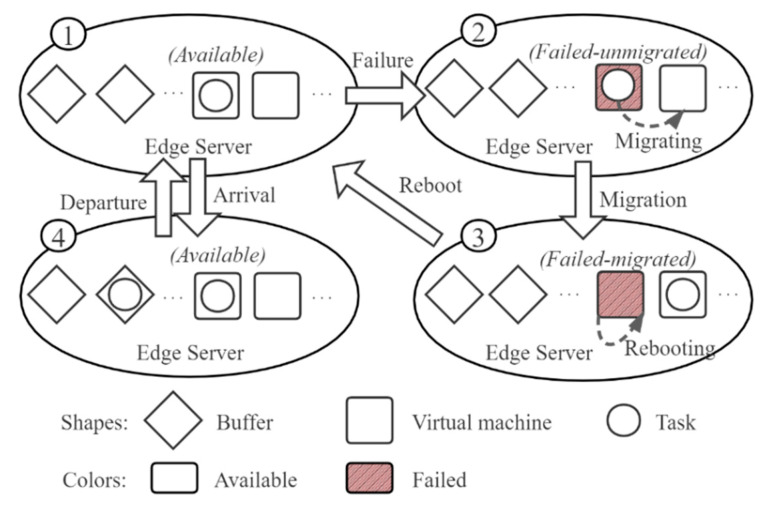
State transitions of virtual machines (VM) in an edge server.

When a VM fails, the task will be migrated to an idle VM and restart the processing, as shown in Figure 2. The migration downtime follows an exponential distribution where the mean service time is 1/α. Note that if all the other VMs are occupied or failed, the task will stay in the failed VM and wait for an idle VM to migrate. Extremely, when all the VMs are failed and not ready to reboot, to avoid deadlock of the VMs, the last failed VM can reboot without migration, reserving the un-migrated task. After the migration of the task, the failed VM can reboot and become an available VM again. If there are fewer than S VMs available (capable to process a task), the newly recovered VM will be assigned as a TVM; otherwise, it will be assigned as a BVM. The reboot time of VM follows an exponential distribution where the mean reboot time is 1/β. Then, each VM has three states: available, failed-unmigrated, and failed-migrated. Here, the available state refers to the state that the VM is capable to process a task. An available VM can be either idle or occupied. Transitions among the three states are depicted in Figure 3: (1) From ① to ②, the running VM fails, transiting from available to failed-unmigrated. (2) If there is at least one idle VM, the migration begins, or the failed VM needs to wait for an idle VM to migrate. When the migration completes, the edge server transits from ② to ③, where the failed VM is failed-migrated. (3) By rebooting, the failed-migrated VM can recover to be available again, edge server transiting from ③ to ①. If there are fewer than S VMs running, the newly recovered VM will be assigned as a TVM, or it will be assigned as a BVM. (4) At ①, when the TVMs are all occupied, the new arrival task will be waiting in the buffer, edge server transiting from ① to ④. If the buffer is full as well, a discard occurs. (5) At ④, if a VM completes the processing of a task and becomes an idle VM, the task waiting in the buffer will get into the idle VM for processing, edge server transiting from ④ to ①.

In the edge computing server described above, the processing of tasks can be treated as a revised M/M/s/K queue, where the VM migration and recovery are considered.

Assumptions:

- All the tasks are equal in data size;
- The failures of VM are all transient failures, which can be recovered by rebooting in a short time;
- Idle VMs do not fail;
- The working times of VMs in the server are independent and identically distributed with the exponential distribution where 1/θ is the mean time to failure.

### 3.2. Analysis of End-to-End Delay

**Figure 4:**
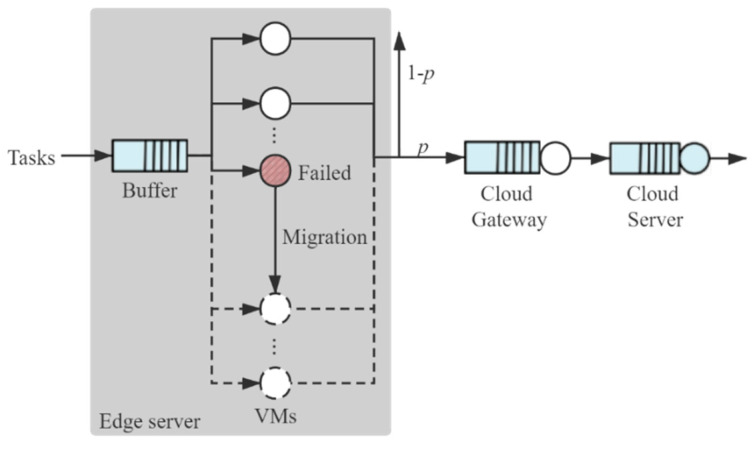
Queueing models through the network.

After the processing at the edge server, a part of task data needs to be transmitted to the cloud for further processing or storage within a delay constraint of $D_{max2}$. According to Burke’s theorem [62], the steady-state departure process of a stable M/M/c queueing system is a Poisson process with the same rate as the arrival process [63]. Assume that the service time of the cloud gateway and the cloud server follow exponential distributions where the mean service time is $1/\mu_{lg}$ and $1/\mu_{cs},$ respectively. Then, the queues at the cloud gateway and the cloud server can be modeled by the M/M/1/K queueing model [64]. As depicted in Figure 4, the tasks are processed at the edge server firstly. Then, a portion p of tasks is transmitted to a cloud server by a cloud gateway for further processing. The delay is caused by the queueing at the edge server, cloud gateway, and cloud server successively.

In the problem, we consider different delay constraints to obtain VTR and ETR of the edge computing network, according to the parameters of the network shown in Table 2.

## 4. Mathematical Model

### 4.1. Queueing Model of the Edge Server

To model the queueing at the VMs in the edge server described in the previous section, we denote the state of the edge server by $\left(x_{1},x_{2},x_{3}\right)$, where integer $x_{1}\in \left[0,Z\right]$ denotes the number of available VMs, integer $x_{2}\in \left[0,Y\right]$ denotes the number of tasks in the edge server, and integer $x_{3}\in \left[0,\min\left(x_{2},\;Z\right)\right]$ denotes the number of failed-unmigrated VMs. Since $x_{1}$, $x_{2}$, and $x_{3}$ are all limited, the number of states is finite. According to Section 3.1, $x_{1}$, $x_{3}$, and $\left(x_{1}+x_{3}\right)$ shall all be less than Z. In addition, the number of tasks $x_{2}$ shall not be more than the present capacity of the edge server $\left[\min\left(x_{1},S\right)+x_{3}+K\right]$. Furthermore, the number of tasks $x_{3}$ reaches its maximum when all the tasks stay in failed-unmigrated VMs, where the maximum value is $x_{2}$. According to the analysis above, the constraints of the three state variables are:

(2) $$\{\begin{array}{c} 0\leq x_{1}\leq Z\\ 0\leq x_{2}\leq \min\left(x_{1},S\right)+x_{3}+K\\ 0\leq x_{3}\leq \min\left(x_{2},\;Z\;-\;x_{1}\right) \end{array}.$$

where Z denotes the total number of VMs, S denotes the number of TVMs, K denotes the capacity of the buffer of the edge server.

We assume that the states violating Equation (2) exist in the CTMC and name them as pseudo-states. The transitions of pseudo-states are the same as the real states. Furthermore, to study the state transitions and express the transition rate diagram conveniently, we divide the states into a few blocks in terms of $x_{2}$, i.e., the states that have the same value of $x_{2}$ belong to the same block. Then the total number of blocks is (Y+1), for $x_{2}$ ranges from 0 to Y. The j-th block $B_{j}$:

(3) $$B_{j}=\left\{\left(x_{1},x_{2},x_{3}\right)|x_{2}=j-1,x_{1}\in \left[0,Z\right],x_{3}\in \left[0,\min\left(x_{2},\;Z\right)\right]\right\}.$$

The number of columns of $B_{j}$. is:

(4) $$a_{j}=\min\left(j,Z\right),\;1\leq j\leq Y+1.$$

Let $b_{j}$ denote the total number of columns from block $B_{1}$ to block $B_{j}$. Then, $b_{j}$ is given by:

(5) $$b_{j}=\sum_{u=1}^{j}a_{u}.$$

**Figure 5:**
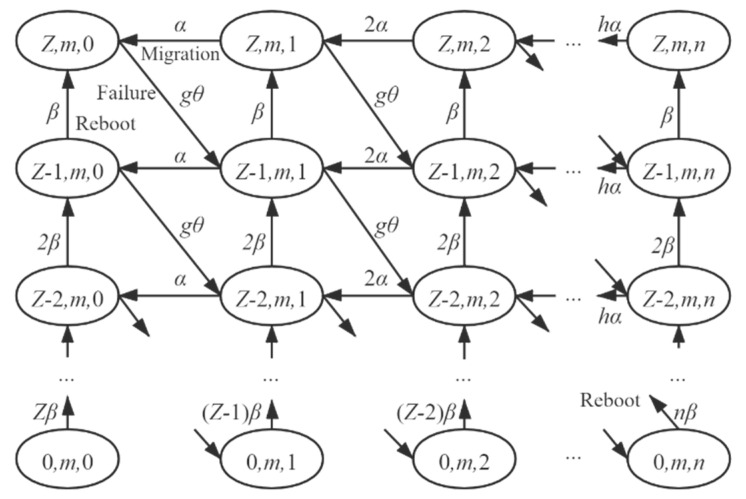
Transitions inside the blocks, triggered by failure (transitions toward bottom-right), migration (leftward transitions), and reboot (upward transitions) of VM. $n=\min\left(x_{2},Z\right)$.

Consequently, the transitions inside a block are all triggered by state transitions of VM (failure, migration, and reboot). Figure 5 shows transitions inside block $B_{m+1}$. There are three kinds of transition:

(1) Transitions caused by failures of VMs. The number of VMs about to fail equals the number of running VMs, which is the minimum of the number of available TVMs $\min\left(x_{1},S\right)$ and the number of live tasks $\left(x_{2}-x_{3}\right)$. Thus, from state $\left(x_{1},x_{2},x_{3}\right)$ to state $\left(x_{1}-1,x_{2},x_{3}+1\right)$, the transition rate is gθ, where g is the number of about-to-fail VMs (or the number of running VMs), given by:

(6) $$g=\min\left(\min\left(x_{1},S\right),x_{2}-x_{3}\right).$$

Notably, the transition of migration does not exist when $x_{1}=0$, since none VM is available.

(2) Transitions caused by migrations of failed VMs. The number of migrating VMs is the minimum of the number of failed-unmigrated VMs $x_{3}$ and the number of idle VMs $\left(x_{1}-g\right)$. Thus, from state $\left(x_{1},x_{2},x_{3}\right)$ to state $\left(x_{1},x_{2},x_{3}-1\right)$, the transition rate is hα, where h is the number of migrating VMs, given by:

(7) $$h=\min\left(x_{3},x_{1}-g\right)=\min\left(x_{3},x_{1}-\min\left(\min\left(x_{1},S\right),x_{2}-x_{3}\right)\right).$$

(3) Transitions caused by the reboot of failed-migrated VMs. The number of rebooting VMs is the number of failed-migrated VMs $\left(Z-x_{1}-x_{3}\right)$. From state $\left(x_{1},x_{2},x_{3}\right)$ to state $\left(x_{1}+1,x_{2},x_{3}\right)$, the transition rate is $\left(Z-x_{1}-x_{3}\right)\beta$.

**Figure 6:**
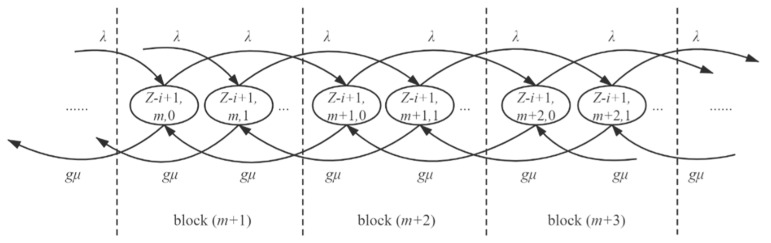
Transitions between blocks when $x_{1}=Z-i+1$, triggered by the arrival (rightward transitions) or departure (leftward transitions) of tasks.

At the same time, transitions between blocks are triggered by the arrival or departure of tasks. Figure 6 presents the transitions between blocks when $x_{1}=Z-i+1$. The presented two states in each block are the first two states of the block. When a task arrives, the k-th state of block $B_{j}$ transits to the k-th state of block $B_{j+1}$ with a transition rate of λ; when a task departs, the k-th state of block $B_{j}$ transits to the k-th state of block $B_{j-1}$ with a transition rate of gμ.

**Figure 7:**
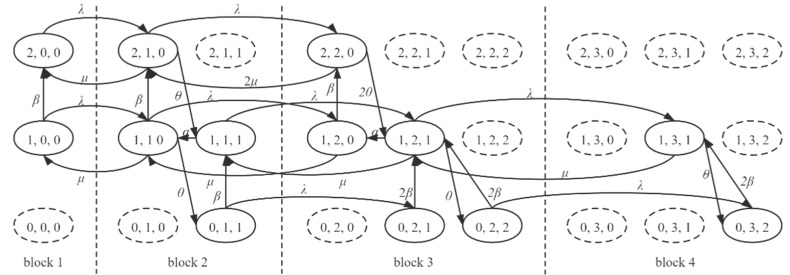
Continuous-time Markov chain (CTMC) of a case where S=1, d=1, K=1. The dashed-line states are pseudo-states.

Note that the transitions inside the blocks and the transitions between the blocks all exist in the CTMC of the edge server. We detach the transitions into two types for a concise and explicit description. Figure 7 presents the states of a case where S=1, d=1, K=1.

Given the partitioned configuration of the CTMC, to locate a state more easily, we define the coordinate of a state as (i,j,k), where i denotes the row number, j denotes the block number, and k denotes the column number inside the block. The derivation between a state and its coordinate is

(8) $$\{\begin{array}{c} x_{1}=Z+1-i\\ x_{2}=j-1\\ x_{3}=k-1 \end{array}.$$

where $x_{1}$ denotes the number of available VMs, $x_{2}$ denotes the number of tasks in the edge server, $x_{3}$ denotes the number of failed-unmigrated VMs. According to Equations (2) and (8), the constraints of the three coordinate variables are:

(9) $$\{\begin{array}{c} 1\leq i\leq Z+1\\ 1\leq j\leq \min\left(Z-i+1,S\right)+k+K\\ 1\leq k\leq \min\left(j,\;i\right) \end{array}.$$

To avoid misunderstanding, we define the set of all the states in row i∈[1,Z+1] of the CTMC as $L_{i}$:

(10) $$L_{i}=\left\{\left(i,j,k\right)|j\in \left[1,Y+1\right],\;k\in \left[1,\min\left(j,\;Z\right)\right]\right\}.$$

Then, in $L_{i}$, the number of states (i,j,k) is $c=b_{j}-a_{j}+k$, where $b_{j}$ denotes the total number of columns from block $B_{1}$ to block $B_{j}$, $a_{j}$ denotes the number of columns of the block $B_{j}$. Since the number of states is numerous, it is hard to give the expression of transition rate for each state if we directly construct the generator matrix according to the sequence of states. Therefore, we construct the generator matrix of the CTMC based on the matrix-geometric method. Specifically, we obtain the generator matrices of transition inside a row $L_{i}$ (transitions caused by arrival and departure of tasks and migration of VMs) and generator matrices of transitions between neighboring rows (transitions caused by failure and reboot of VMs), respectively. In this way, we can express the generator matrix as a partitioned matrix with quasi-birth-and-death feature:

(11) $$A=\left[\begin{array}{cccccc} A_{11} & A_{12} & 0 & 0 & 0 & 0\\ A_{21} & A_{22} & A_{23} & 0 & 0 & 0\\ 0 & A_{32} & A_{33} & \ddots & \vdots & \vdots \\ \vdots & 0 & \ddots & \ddots & A_{Z-1,Z} & 0\\ 0 & \vdots & 0 & A_{Z,Z-1} & A_{Z,Z} & A_{Z,Z+1}\\ 0 & 0 & 0 & 0 & A_{Z+1,Z} & A_{Z+1,Z+1} \end{array}\right],$$

where matrix $A_{xy_{\left(b_{Y+1}\times b_{Y+1}\right)}}$ denotes the generator matrix from $L_{x}$ to $L_{y}$. Then, the element $A_{xy}\left(c_{1},c_{2}\right)$ in row $c_{1}$ and column $c_{2}$ of matrix $A_{xy}$ denotes the transition rate from the $c_{1}$-th state of $L_{x}$ to the $c_{2}$-th state of $L_{y}$. The general form of $A_{xy}$ is given by:

(12) $$\{\begin{array}{c} A_{ii}\left(b_{j}-a_{j}+k,b_{j+1}-a_{j+1}+k\right)=N\lambda ,1\leq i\leq Z+1,\;1\leq j\leq Y,\;1\leq k\leq \min\left(j,Z\right)\\ A_{ii}\left(b_{j+1}-a_{j+1}+k,b_{j}-a_{j}+k\right)=g\mu ,1\leq i\leq Z+1,\;1\leq j\leq Y,\;1\leq k\leq \min\left(j,Z\right)\\ A_{ii}\left(b_{j}-a_{j}+k,b_{j}-a_{j}+k-1\right)=h\alpha ,1\leq i\leq Z+1,\;2\leq j\leq Y+1,\;2\leq k\leq \min\left(j,Z\right)\\ A_{i,i+1}\left(b_{j}-a_{j}+k,b_{j}-a_{j}+k+1\right)=g\theta ,\;1\leq i\leq Z,\;2\leq j\leq Y+1,\;1\leq k\leq \min\left(j,Z\right)-1\\ A_{i+1,i}\left(b_{j}-a_{j}+k,b_{j}-a_{j}+k\right)=i\beta ,\;1\leq i\leq Z,\;1\leq j\leq Y+1,\;1\leq k\leq \min\left(j,Z\right)\\ A_{i+1,i}\left(b_{j}-a_{j}+k+1,b_{j}-a_{j}+k\right)=\left(k-1\right)\beta ,i=Z+1,\;Z+1\leq j\leq Y+1,\;k=Z+1 \end{array},$$

where g is the number of running VMs, h is the number of migrating VMs.

Identify the pseudo-states according to Equation (2). Then, delete the rows and columns of the pseudo-states in A and name the remaining l×l matrix as Q. After that, revise the diagonal elements of Q to subject to:

(13) $$Q_{l\times l}\cdot 1_{l\times 1}=0_{l\times 1}.$$

The matrix Q is the final form of the generator matrix of the CTMC.

To obtain the PDF of sojourn time at the edge server, we employ the first passage time method. Among all the states of the edge computing server, the states in block 1 represent that the edge server is empty, for the numbers of the tasks are 0. Delete the states in block 1. Then, the sojourn time at the edge server approximately equals the time that the edge server transits from the non-empty states to the empty states when no new task arrives, which can be obtained by the first passage time method. Obtaining the PDF of sojourn time at the edge server follows the steps below:

1. Solve Equation (14) to obtain the steady-state probability vector π of the CTMC:

(14) $$\{\begin{array}{c} Q^{T}\pi^{T}=0\\ \sum^{\;}\pi =1 \end{array};$$

2. Delete the empty states in the transition rate matrix A, and retain the rest states. The new matrix is named as M.

3. Delete the probability of empty states in the stable probability vector π. The new vector is named as γ. Let $\delta =\gamma /\sum^{\;}\gamma$.

4. The distribution function of sojourn time D is:

(15) $$F_{es}\left(t\right)=P\left(D\leq t\right)=1-\delta \cdot \exp\left(Mt\right)\cdot 1.$$

5. Then PDF of the sojourn time D is:

(16) $$f_{es}\left(t\right)=F_{es}^{\prime }\left(t\right).$$

### 4.2. Timely Reliability Model

As analyzed in Section 3, the queueing at the cloud gateway and cloud server can be modeled by the M/M/1/C model. The tasks go through the queue of the edge server, the queue of cloud gateway, and the queue of the cloud server successively. Then, the end-to-end delay is determined by the joint distribution of the successive delays of a task traversing edge server, cloud gateway, and cloud server. The PDF of delay at the VMs in the edge server has been analyzed in Section 4.1. In the rest of Section 4.2, we present the analysis method of end-to-end delay and timely reliability, including VTR and ETR.

#### 4.2.1. VMs Timely Reliability

VMs timely reliability (VTR) is defined as the probability that the VMs in an edge server are capable of processing a task within a required time. For tasks requiring edge service, the service delay equals the sojourn time at the edge server. According to Equation (1), VTR is:

(17) $$R_{V}=\int_{0}^{D_{max1}}f_{es}\left(t\right)dt,$$

where $D_{max1}$ is the maximum allowable delay at the edge server.

#### 4.2.2. End-to-End Timely Reliability

End-to-end timely reliability (ETR) is defined as the probability that a group of end-to-end nodes in the edge computing network is capable of processing a task within a required time. For tasks requiring cloud service, the service delay is the sum of delay at the edge server, cloud gateway, and cloud. Since only a portion p of tasks is transmitted to the cloud server, the arrival rate of tasks at the cloud gateway and the cloud server is pNλ. Departing from the edge server, a task arrives at the cloud gateway finding w tasks ($0\leq w\leq C_{lg}$) already there with a probability [24]:

(18) $$P_{lg}^{w}=\{\begin{array}{c} \frac{\left(1-\rho_{lg}\right){\left(\rho_{lg}\right)}^{w}}{1-\rho_{lg}{}^{C_{lg}}}\;,\rho_{lg}\neq 1\\ \frac{1}{C_{lg}}\;,\rho_{lg}=1 \end{array},$$

where $\rho_{lg}=pN\lambda /\mu_{lg}$ is the traffic intensity at the cloud gateway. The processing time of a task at the cloud gateway follows exponential distribution:

(19) $$f_{lg}^{0}\left(t\right)=\mu_{lg}e^{-\mu_{lg}t},\;t>0.$$

Then, the sojourn time of a task is the joint distribution of the processing time of all the (w+1) tasks (the former w tasks and itself). Since the sum of identically and exponentially distributed variables follow a Gamma distribution, the PDF of the sojourn time of a task at the cloud gateway if it arrives at the cloud gateway finding w tasks already there is:

(20) $$f_{lg}^{w}\left(t\right)=\frac{\mu_{lg}{}^{w}}{w!}\cdot t^{w-1}\cdot e^{-\mu_{lg}t},\;t>0.$$

Therefore, the PDF of the sojourn time at the cloud gateway:

(21) $$f_{lg}\left(t\right)=\sum_{w=0}^{C_{lg}-1}P_{lg}^{w}\cdot f_{lg}^{w}\left(t\right).$$

Similarly, departing from the cloud gateway, a task arrives at the cloud server finding w tasks ($0\leq w\leq C_{cs}$) already there with a probability:

(22) $$P_{cs}^{w}=\{\begin{array}{c} \frac{\left(1-\rho_{cs}\right){\left(\rho_{cs}\right)}^{w}}{1-{\left(\rho_{cs}\right)}^{C_{cs}}},\;\rho_{cs}\neq 1\\ \frac{1}{C_{cs}},\;\rho_{cs}=1 \end{array},$$

where $\rho_{cs}=pN\lambda /\mu_{cs}$ is the traffic intensity at the cloud server. The sojourn time of a task at the cloud server if it arrives at the cloud server finding w tasks already there is:

(23) $$f_{cs}^{w}\left(t\right)=\frac{\mu_{cs}{}^{w}}{w!}\cdot t^{w-1}\cdot e^{-\mu_{cs}t},\;t>0.$$

The PDF of the sojourn time at the cloud server:

(24) $$f_{cs}\left(t\right)=\sum_{w=0}^{C_{cs}-1}P_{cs}^{w}\cdot f_{cs}^{w}\left(t\right).$$

According to the above analysis, PDF of the end-to-end delay of the edge computing network can be obtained by:

(25) $$f\left(t_{3}\right)=\int_{0}^{+\infty }\left[\int_{0}^{+\infty }f_{es}\left(t_{1}\right)f_{lg}\left(t_{2}-t_{1}\right)dt_{1}\right]f_{cs}\left(t_{3}-t_{2}\right)dt_{2},$$

where $t_{1}$, $t_{2}$, and $t_{3}$ are the sojourn time at the edge server, the total time to go through the edge server, and the cloud gateway, and the total time to go through the edge server, the cloud gateway, and the cloud server, respectively. According to Equation (1), ETR can be obtained by:

(26) $$R_{N}=\int_{0}^{D_{max2}}f\left(t_{3}\right)dt_{3}.$$

### 4.3. Algorithm

To calculate VTR, the key is to solve the CTMC with a state-space of high dimension and obtain the PDF of the sojourn time at the edge server. For this purpose, we need to propose an algorithm to formulate the partitioned generator matrix and obtain the PDF of the sojourn time at the edge server based on the first passage time method. The obtaining of PDF of the sojourn time at the edge server is described in Algorithm 1. After that, VTR and ETR can be calculated according to Section 4.2.
**Algorithm1** Algorithm for obtaining PDF of the sojourn time at the edge server    **Input:**            Arrival rate of tasks: λ            Number of sensors: N            Service rate of VM: μ            Number of TVMs: S            Number of BVMs: d            Capacity of the buffer: K            Failure rate of VMs: θ            Migration rate of VMs: α            Reboot rate of VMs: β    **Output:**            PDF of the sojourn time at the edge server: $f_{es}\left(t\right)$
1. **initialize**
A
2. vector a=min(j,Z), 1≤j≤Y+1 % The number of columns of block j
3. vector $b=\sum_{u=1}^{j}a_{u},\;1\leq j\leq Y+1$ % The number of total columns from block 1 to block j
4. **for**
*i* = 1: *Z* + 1 **do** % Transitions caused by arrival or departure of tasks
5.     **for**
*j* = 1: *Y*
**do**
6.         **for**
*k* = 1: min(a(*j*), *i*) **do**
7.             *x* = b(*j*) − a(*j*) + *k*
8.             *y* = b(*j* + 1) − a(*j* + 1) + *k*
9.             A{i, i} (x, y)=Nλ
10.           A{i, i} (*y*, *x*) = min (min (*Z* + 1 − *i*, *S*), *j* − *k*) * μ
11.       **end for**
12.     **end for**
13. **end for**
14. **for**
*i* = 1: *Z* + 1 **do** % Transitions caused by migration of VMs
15.     **for**
*j* = 2: *Y* + 1 **do**
16.         **for**
*k* = a(*j*): −1: 2 **do**
17.             *x* = b(*j*) − a(*j*) + *k*
18.             A{*i*, *i*} (*x*, *x* − 1) = min (*k* − 1, *Z* + 1 − *i* − min (min (*Z* + 1 − *i*, *S*), *j* − *k*)) * α
19.         **end for**
20.     **end for**
21. **end for**
22. **for**
*j* = 2: *Y* + 1 **do** % Transitions caused by migration of VMs
23.     **for**
*k* = 1: a(*j*) − 1 **do**
24.         **for**
*i* = 1: *Z*
**do**
25.             x=b(j)−a(j)+k
26.             A {i, i+1} (x, x+1) = min (min (*Z* + 1 − *i*, S), *j* − *k*) * θ
27.         **end for**
28.     **end for**
29. **end for**
30. **for**
*j* = 1: *Y* + 1 **do** % Transitions caused by reboot of VMs
31.     **for**
*k* = 1: a(*j*) **do**
32.         **for**
*i* = 2: *Z* + 1 **do**
33.             *x* = b(*j*) − a(*j*) + *k*
34.             A{*i*, *i* − 1} (*x*, *x*) = *i*β
35.         **end for**
36.     **end for**
37. **end for**
38. **for**
*j* = *Z* + 1: *Y* + 1 **do** % Transitions caused by reboot without migration when all the VMs are failed-unmigrated
39.     *k* = a(*j*)
40.     *x* = b(*j*) − a(*j*) + *k*
41.     A{*Z* + 1, *Z*} (*x*, *x*−1) = (*k* − 1) * β
42. **end for**
43. Q = A
44. **for**
*j* = 1: size (a,2) **do** % Identify pseudo-states
45.     **for**
*k* = 1: a(*j*) **do**
46.         **for**
*i* = 1: *Z* + 1 **do**
47.             **if**
k≤min(j, i)|| j≤min(Z−i+1,S)+k+K
48.                 *x* = (*i* − 1) * b(*Y* + 1) + b(*j*) − a(*j*) + *k*
49.                 replace row *x* of Q with **0**
50.                 replace column *x* of Q with **0**
51.             **end if**
52.         **end for**
53.     **end for**
54. **end for**
55. replace the rows and columns of absorbing states with **0**
56. record the row number (or column number) of the states whose value is **0**
57. revise the diagonal elements of Q to ensure the sum of each row equal to 0
58. delete the rows and columns whose value is **0**
59. the steady-state probability vector: π=Q/e %e is a column vector whose last element is 1, and the other elements are 0
60. M = A
61. set the arrival rate to 0 in M
62. revise the diagonal elements of M to ensure the sum of each row equals to 0
63. delete the rows and columns of the states of block 1 % The states of block 1 represent that the edge server is empty
64. delete the rows and columns that equals to **0**
65. delete the probability of empty states in the stable probability vector π and name the new vector as γ
66. let $\delta =\gamma /\sum^{\;}\gamma$
67. $F_{es}\left(t\right)=1-\delta \cdot \exp\left(Mt\right)\cdot 1$
68. calculate the PDF of sojourn time at the edge server: $f_{es}\left(t\right)=F_{es}^{\prime }\left(t\right)$
69. return $f_{es}\left(t\right)$

## 5. Results and Discussion

In this section, we provide a case study of traffic monitoring for the proposed timely reliability evaluation method. Simulation results are obtained to verify our model and numerical results. We also investigate the influence of different factors on timely reliability, which helps design a better edge computing network.

### 5.1. Experimental Setup

At a crossroads, the edge computing network for traffic monitoring consists of several sensors, an edge server, a cloud gateway, and a cloud server. The architecture of this network is depicted in Figure 1. To monitor the traffic at this crossroads, the sensors collect task data in real-time and transmit the tasks to the edge server, where the tasks are processed by VMs. In addition, a part of processed tasks is transmitted to the cloud server through the cloud gateway for further process or storage. The parameters used in this case are shown in Table 3. The data on sensor data refer to [65,66]. The data on VM migration refer to [40]. The data on system failure refer to [10].

### 5.2. Simulation Experiments

**Figure 8:**
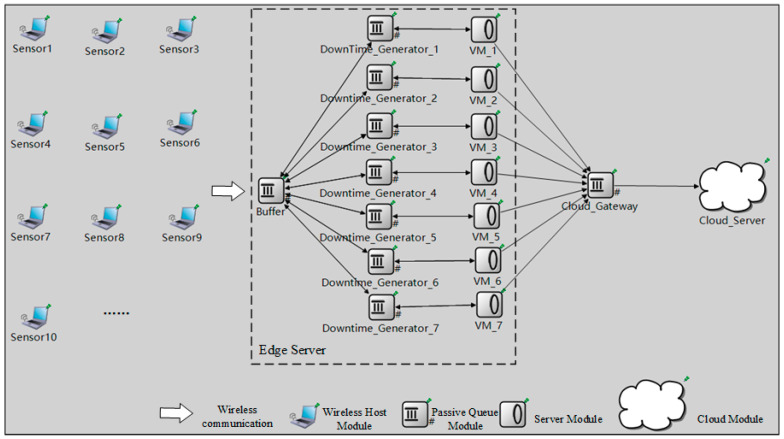
Simulation model in OMNeT++.

The numerical results are verified by simulation based on OMNeT++ (V5.5.1), a C++ network simulation library and framework. We choose OMNeT++ for its open source and extensible features. Figure 8 shows the simulation model in OMNeT++. In the simulation model, the sensors are modeled by the Wireless Host Module; the buffer of the edge server is modeled by the Passive Queue Module; the VMs of the edge server are modeled by the Server Module; the cloud gateway is modeled by the Passive Queue Module; the cloud server is modeled by the Cloud Module. Particularly, to model the migration time of the VM migration, a Downtime Generator (DG), modeled by the Passive Queue Module, is employed to connect the edge servers and VMs. Once a running VM fails, the task on this VM will be migrated to an idle VM through the DG, where a migration downtime of an exponential distribution is needed. Notably, the tasks spend no time to pass the DG unless it is on the way of migration. As soon as the migrating task arrives at the destination VM, the failed VM becomes failed-migrated, and its rebooting can begin. The values of the parameters are the same as those in Table 3.

**Figure 9:**
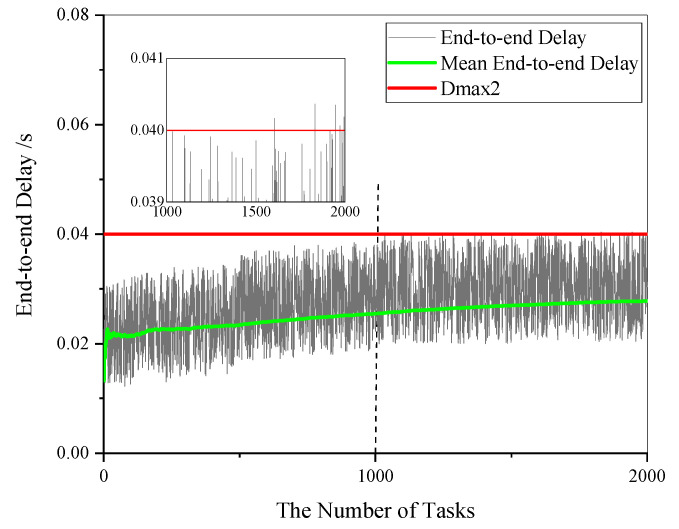
Converging of the end-to-end delay in a simulation.

In the simulation, the end-to-end delay converges to a steady-state value as the simulation time goes by, as shown in Figure 9. We collect the delay values of each 1000 tasks to calculate ETR. When the variation of ETR is less than 1%, we take the final value as the stationary simulation result. Let the Boolean variable $F_{i}$ denote if the end-to-end delay of a task satisfies the task requirement:

(27) $$F_{i}=\{\begin{array}{c} 0,\;\mathrm{the}\;\mathrm{delay}\;\mathrm{does}\;\mathrm{not}\;\mathrm{satisfy}\;\mathrm{the}\;\mathrm{requirement}\;D_{max2}\\ 1,\;\mathrm{the}\;\mathrm{delay}\;\mathrm{satisfies}\;\mathrm{the}\;\mathrm{requirement}\;\;D_{max2} \end{array}.$$

Then ETR can be obtained by:

(28) $$R_{N}=\frac{\sum_{i=1}^{n}F_{i}}{n}.$$

**Figure 10:**
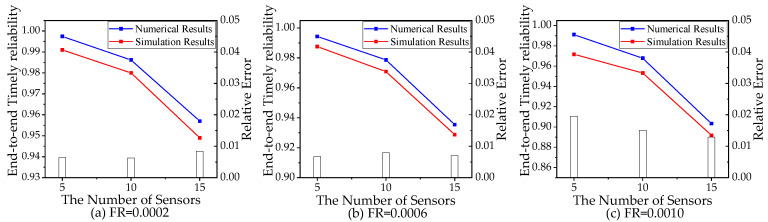
Numerical results and simulation results with failure rate (FR) equals (**a**) 0.0002, (**b**) 0.0006, and (**c**) 0.0010.

In the simulation, it takes 5 min for the end-to-end delay to converge. To obtain the steady-state value of ETR, the simulation takes more than 10 min. We compare the simulation results and the numerical results of ETR when the failure rate θ equals to 0.0002, 0.0006, and 0.001 with different numbers of sensors. The comparisons are shown in Figure 10, where the bars’ heights represent the relative errors of the numerical results. The relative errors are all less than 2%. The simulation results verify that the analytical model proposed in this paper is valid and applicable for timely reliability analysis of a computing network considering failure, migration, and reboot of edge server VMs.

### 5.3. Timely Reliability Analysis

To investigate the impacts of different factors on the timely reliability of the VMs, we compare VTR under different conditions based on the analysis methods we proposed.

#### 5.3.1. Analysis of Failure Rate, Migration Rate, and Reboot Rate

**Figure 11:**
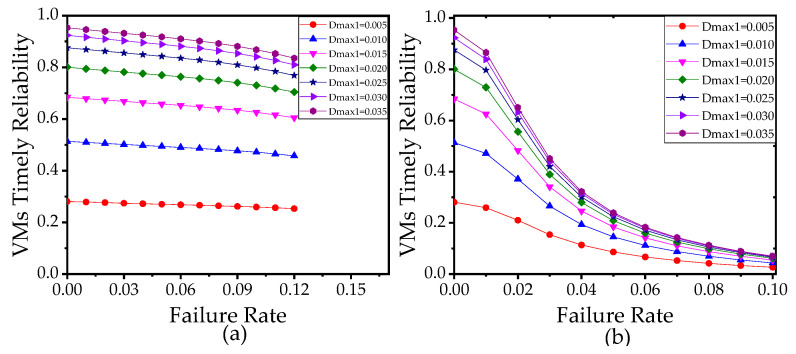
(**a**) VTR versus the failure rate with different maximum allowable edge delay $D_{max1}$, when the migration rate α = 0.002, the reboot rate β = 100α. (**b**) VTR versus the failure rate with different $D_{max1}$, when the migration rate α = 0.002, the reboot rate β = 100α. In (**a**,**b**), the number of sensors is 10, the failure rate of VMs θ = 0.0002. The other parameters used in (**a**,**b**) refer to Table 3.

**Figure 12:**
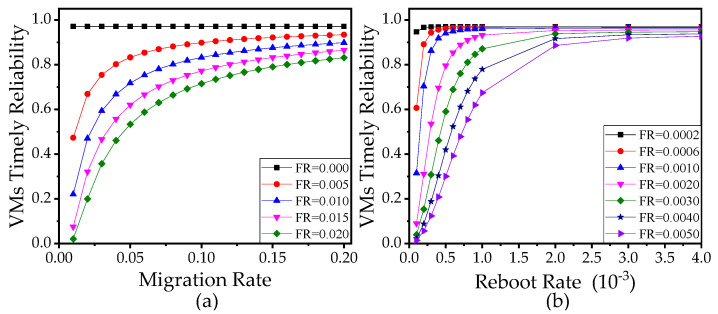
(**a**) VTR versus the migration rate with different failure rates (FR). (**b**) VTR versus the reboot rate with different failure rates (FR). In (**a**,**b**), the number of sensors is 10. The other parameters used in (**a**,**b**) refer to Table 3.

It is observed in Figure 11 and Figure 12 that VTR is a monotonic function of the failure rate, migration rate, and reboot rate of VM. Comparing Figure 11a with Figure 11b, we find VTR is more sensitive to the failure rate when the reboot rate and migration rate are smaller since the VMs spend more time to migrate and recover when the reboot rate and migration rate are smaller. Thus, if the migration time and reboot time are quite short, the designers do not need to be very rigid on the reliability of VMs. Conversely, if the migration time and reboot time are relatively long, designers can improve VTR of the edge computing network effectively by reducing the failure rate of VMs.

#### 5.3.2. Analysis of the Number of VMs

**Figure 13:**
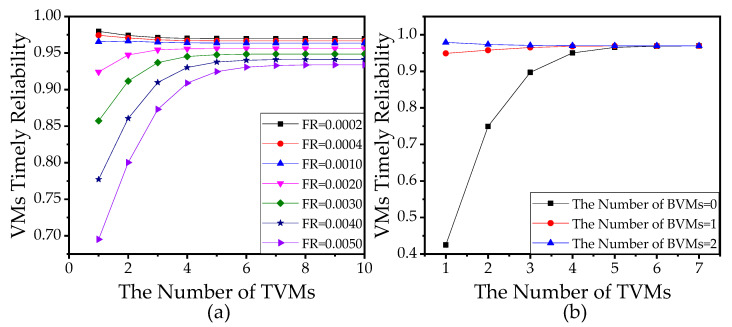
(**a**) VTR versus the number of task VMs (TVMs) with different failure rate (FR). The number of backup VMs (BVM) d=2. (**b**) VTR versus the number of TVMs with different numbers of BVMs where the failure rate θ=0.0002. In (**a**,**b**), the number of sensors is 10 and the total number of VMs is not fixed. The other parameters used in (**a**,**b**) refer to Table 3.

The number of TVMs and BVMs are also influential factors of VTR. Results in Figure 13a show that VTR is higher with the increase in the number of TVMs when the failure rate is high. The reason is that more VMs mean the server can process more tasks at the same time. However, when the failure rate is low, we have an interesting finding that VTR monotonically decreases as the number of TVMs grows, such as the cases when the failure rate equals 0.0002 or 0.0004 in Figure 13a. In fact, the increase in the number of TVMs influences VTR by two means, i.e., (1) reducing the end-to-end delay by processing more tasks at the same time and (2) increasing the end-to-end delay by bringing about more failure, migration, and reboot of VMs in a given time. The second impact is more significant when the failure rate is high. Whereas when the failure rate is low, the first impact is more significant. Similar results are shown by Figure 13b, where with no BVM or extremely few BVMs, VTR monotonically increases as the number of TVMs grows, indicating that the first impact is more significant. However, with more BVMs, VTR monotonically decreases as the number of TVMs grows, indicating that the second impact is more significant. Therefore, the increase in TVMs is not always a good choice for improving VTR.

**Figure 14:**
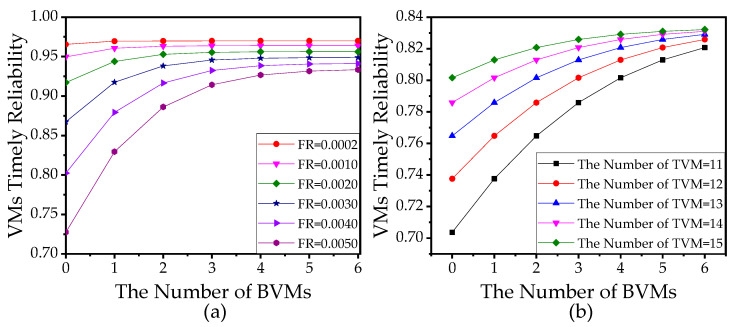
(**a**) VTR versus the number of backup VMs (BVM) with different failure rates (FR). (**b**) VTR versus the number of BVMs with different numbers of task VMs (TVMs) where the failure rate θ=0.0002. In (**a**,**b**), the number of sensors is 10. The other parameters used in (**a**,**b**) refer to Table 3.

According to the above findings, the resource manager can allocate an appropriate number of TVMs according to the above-mentioned analysis, which can be a guidance for the initial configuration of VMs. As for the number of BVMs, Figure 14 shows that VTR monotonically increases as the number of BVM grows.

#### 5.3.3. Management of VMs

**Figure 15:**
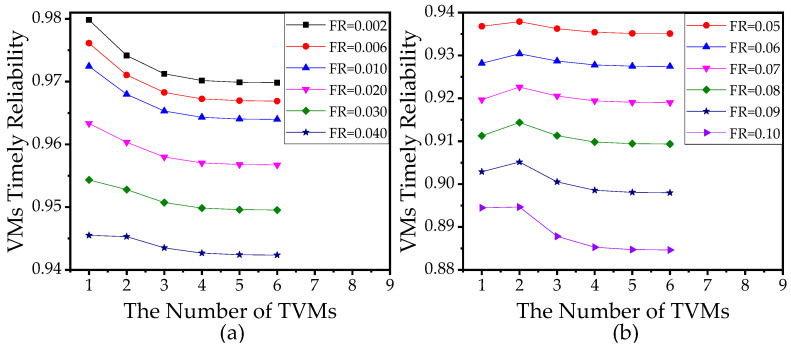
(**a**) VTR versus the number of task VMs (TVMs) where the total number of VMs is 10 and the failure rate (FR) ranges from 0.002 to 0.04. (**b**) VTR versus the number of TVMs where the total number of VMs is 10 and the FR ranges from 0.05 to 0.1. In (**a**,**b**), the number of sensors is 10. The other parameters used in (**a**,**b**) refer to Table 3.

Since the increase in the number of TVMs and the number of BVMs both improve VTR generally, to achieve the highest VTR, there is a trade-off between the number of TVMs and the number of BVMs when the total number of VMs is limited. Figure 15a reveals that when the failure rate of VM is low, only assigning one VM as TVM is the best solution. Figure 15b shows that when the failure rate is high, more VMs should be assigned as TVMs to achieve the highest VTR. Therefore, to achieve the highest VTR, based on the analysis method of this paper, the resource manager can find the optimal allocation of VMs according to the related parameters of the edge computing network and the tasks.

### 5.4. Section Summary

In this section, we provide a case study of traffic monitoring for the proposed timely reliability evaluation method and introduce the simulation method based on OMNeT++. Simulation results verify that the analytical model proposed in this paper is valid and applicable for timely reliability analysis of VMs of an edge server, considering failure, migration, and reboot of VMs.

We also investigate the influence of different factors on timely reliability. Results show that: (1) VTR is a monotonic function of the failure rate, migration rate, and reboot rate of VM and is more sensitive to the failure rate when the reboot rate and migration rate are smaller. (2) VTR monotonically increases as the number of BVM grows, but the increase in TVMs does not always improve VTR. (3) To achieve the highest VTR, there is a trade-off between the number of TVMs and the number of BVMs when the total number of VMs is limited.

## 6. Conclusions

In this paper, we analyzed the VTR and the ETR of the edge computing network, considering the failure, migration, and reboot of VMs. To model the state transitions of VMs, we define three states of VMs (available, fail-unmigrated, fail-migrated) and construct a partitioned CTMC for the edge server. We express the generator matrix as a partitioned matrix based on the matrix-geometric method. An algorithm is proposed to resolve the CTMC and obtain the sojourn time at the edge server as well as VTR. Moreover, to analyze ETR of the network, we build the delay analysis model based on Burke’s theorem and propose an end-to-end delay analysis method. The proposed method is more eligible to model the multi-state system of VMs. When the dimension of CTMC states is high, the constructing method of the generator matrix proposed in this paper can effectively reduce the difficulty of algorithm designing.

We investigate VTR of the edge computing network under different conditions. The numerical results are verified to be correct by simulation based on OMNeT++. Results show that VTR is a monotonic function of the migration rate, reboot rate of VM, the number of TVMs, and the number of BVMs. However, in some cases, the increase in TVMs may conversely decrease VTR, since more TVMs also bring about more failures in a given time. Moreover, we find that there is a trade-off between the number of TVMs and the number of BVMs when the total number of VMs is limited. The resource manager can find the optimal allocation of VMs according to the migration rate, reboot rate, failure rate, etc., of the edge computing network and the arrive rate, computation complexity, etc., of the tasks. Based on the trade-off of TVMs and BVMs, in future research, we will further study the optimization of computing resources to obtain a better performance of the edge server. We will also study the case when the sizes of task data are different in future work.

## Figures and Tables

**Table 2:** Notations.

Notation	Description
λ	Arrival rate of tasks
N	Number of sensors
μ	Service rate of virtual machine (VM)
S	Number of task VMs (TVMs)
d	Number of backup VMs (BVMs)
K	Capacity of the buffer of the edge server
Z	Total number of VMs
Y	Maximum capacity of the edge server
θ	Failure rate of VM
α	Migration rate of VM
β	Reboot rate of VM
p	The proportion of tasks requiring cloud service
$\mu_{lg}$	Service rate of cloud gateway
$C_{lg}$	Capacity of cloud gateway
$\mu_{cs}$	Service rate of cloud server
$C_{cs}$	Capacity of cloud server
$D_{max1}$	Maximum allowable delay at the edge server
$D_{max2}$	Maximum allowable end-to-end delay
$x_{1}$	Number of available VMs
$x_{2}$	Number of tasks in the edge server
$x_{3}$	Number of failed-unmigrated VMs
i	State row number
j	State block number
k	State column number inside the block
g	Number of about-to-fail VMs
h	Number of migrating VMs
$B_{w}$	The w-th block of the CTMC
$L_{w}$	Set of all the states in row w of the CTMC

**Table 3:** Inputting parameters of simulation experiments.

Parameter	Description	Value
λ	Arrival rate of tasks	180 per s
N	Number of sensors	10
μ	Service rate of VM	1000 per s
S	Number of TVMs	5
d	Number of BVMs	2
K	Capacity of the buffer of the edge server	180
Z	Total number of VMs	7
Y	Maximum capacity of the edge server	187
θ	Failure rate of VM	0.0002 per s
α	Migration rate of VM	0.2 per s
β	Reboot rate of VM	0.002 per s
p	The proportion of tasks requiring cloud service	1%
$\mu_{lg}$	Service rate of cloud gateway	200 per s
$C_{lg}$	Capacity of cloud gateway	100
$\mu_{cs}$	Service rate of cloud server	300 per s
$C_{cs}$	Capacity of cloud server	100
$D_{max1}$	Maximum allowable delay at the edge server	0.04 s
$D_{max2}$	Maximum allowable end-to-end delay	0.04 s

## Data Availability

The data presented in this study are available in the supplementary material, Data.zip.

## Supplementary Materials

The following are available online at https://www.mdpi.com/1424-8220/21/1/93/s1.

## References

[1] Hassan S.R., Ahmad I., Ahmad S., Alfaify A., Shafiq M. (2020). Remote Pain Monitoring Using Fog Computing for e-Healthcare: AnEfficient Architecture. Sensors.

[2] Qadri Y.A., Nauman A., Zikria Y.B., Vasilakos A.V., Kim S.W. (2020). The Future of Healthcare Internet of Things: A Survey of Emerging Technologies. IEEE Commun. Surv. Tutor..

[3] Osanaiye O., Chen S., Yan Z., Lu R., Choo K.-K.R., Dlodlo M. (2017). From Cloud to Fog Computing: A Review and a Conceptual Live VM Migration Framework. IEEE Access.

[4] Tao Z., Xia Q., Hao Z., Li C., Ma L., Yi S., Li Q. (2019). A Survey of Virtual Machine Management in Edge Computing. Proc. IEEE.

[5] Jennings B., Stadler R. (2015). Resource Management in Clouds: Survey and Research Challenges. J. Netw. Syst. Manag..

[6] Grigorescu S., Cocias T., Trasnea B., Margheri A., Lombardi F., Aniello L. (2020). Cloud2Edge Elastic AI Framework for Prototyping and Deployment of AI Inference Engines in Autonomous Vehicles. Sensors.

[7] Zhang F., Liu G., Fu X., Yahyapour R. (2018). A Survey on Virtual Machine Migration: Challenges, Techniques, and Open Issues. IEEE Commun. Surv. Tutor..

[8] Nkenyereye L., Nkenyereye L., Adhi Tama B., Reddy A.G., Song J. (2020). Software-Defined Vehicular Cloud Networks: Architecture, Applications and Virtual Machine Migration. Sensors.

[9] Garraghan P., Townend P., Xu J. An Empirical Failure-Analysis of a Large-Scale Cloud Computing Environment. Proceedings of the 2014 IEEE 15th International Symposium on High-Assurance Systems Engineering.

[10] Xu J., Kalbarczyk Z., Iyer R.K. Networked Windows NT system field failure data analysis. Proceedings of the 1999 Pacific Rim International Symposium on Dependable Computing.

[11] Bernstein P.A., Newcomer E. (2009). System Recovery, In Principles of Transaction Processing.

[12] Voorsluys W., Broberg J., Venugopal S., Buyya R., Hutchison D., Kanade T., Kittler J., Kleinberg J.M., Mattern F., Mitchell J.C., Naor M., Nierstrasz O., Pandu Rangan C., Steffen B. (2009). Cost of Virtual Machine Live Migration in Clouds: A Performance Evaluation. Cloud Computing.

[13] Noshy M., Ibrahim A., Ali H.A. (2018). Optimization of live virtual machine migration in cloud computing: A survey and future directions. J. Netw. Comput. Appl..

[14] Zhang J., Ren F., Lin C. Delay guaranteed live migration of Virtual Machines. Proceedings of the IEEE INFOCOM 2014-IEEE Conference on Computer Communications.

[15] Li S., Huang N., Chen J., Kang R. Analysis for application reliability parameters of communication networks. Proceedings of the 2012 International Conference on Quality, Reliability, Risk, Maintenance, and Safety Engineering.

[16] Zhao F., Huang N., Chen J.X. (2013). Impact Analysis of Communication Network Reliability Based on Node Failure. Appl. Mech. Mater..

[17] Babay A., Wagner E., Dinitz M., Amir Y. Timely, Reliable, and Cost-Effective Internet Transport Service Using Dissemination Graphs. Proceedings of the 2017 IEEE 37th International Conference on Distributed Computing Systems (ICDCS).

[18] Li R., Li M., Liao H., Huang N. (2017). An efficient method for evaluating the end-To-End transmission time reliability of a switched Ethernet. J. Netw. Comput. Appl..

[19] Zurawski R. (2005). The Industrial Information Technology Handbook.

[20] Liou C.-D. (2015). Markovian queue optimisation analysis with an unreliable server subject to working breakdowns and impatient customers. Int. J. Syst. Sci..

[21] Yang D.-Y., Wu Y.-Y. (2017). Analysis of a finite-capacity system with working breakdowns and retention of impatient customers. J. Manuf. Syst..

[22] Jiang T., Xin B., Chang B., Liu L. (2018). Analysis of a queueing system in random environment with an unreliable server and geometric abandonments. RAIRO-Oper. Res..

[23] Chakravarthy S.R., Shruti, Kulshrestha R. (2020). A queueing model with server breakdowns, repairs, vacations, and backup server. Oper. Res. Perspect..

[24] El Kafhali S., Salah K. (2017). Efficient and dynamic scaling of fog nodes for IoT devices. J. Supercomput..

[25] Pereira P., Araujo J., Torquato M., Dantas J., Melo C., Maciel P. (2020). Stochastic performance model for web server capacity planning in fog computing. J. Supercomput..

[26] Ke J.-C., Lin C.-H., Yang J.-Y., Zhang Z.G. (2009). Optimal (d, c) vacation policy for a finite buffer M/M/c queue with unreliable servers and repairs. Appl. Math. Model..

[27] Liu C.-F., Bennis M., Poor H.V. Latency and Reliability-Aware Task Offloading and Resource Allocation for Mobile Edge Computing. Proceedings of the 2017 IEEE Globecom Workshops (GC Wkshps).

[28] Xie M., Haenggi M. (2009). Towards an end-To-End delay analysis of wireless multihop networks. Ad. Hoc. Netw..

[29] Wang B., Chang X., Liu J. (2015). Modeling Heterogeneous Virtual Machines on IaaS Data Centers. IEEE Commun. Lett..

[30] Chang X., Wang B., Muppala J.K., Liu J. (2016). Modeling Active Virtual Machines on IaaS Clouds Using an M/G/m/m+K Queue. IEEE Trans. Serv. Comput..

[31] Li L., Guo M., Ma L., Mao H., Guan Q. (2019). Online Workload Allocation via Fog-Fog-Cloud Cooperation to Reduce IoT Task Service Delay. Sensors.

[32] Huang N., Chen Y., Hou D., Xing L., Kang R. (2011). Application reliability for communication networks and its analysis method. J. Syst. Eng. Electron..

[33] Yousefpour A., Ishigaki G., Gour R., Jue J.P. (2018). On Reducing IoT Service Delay via Fog Offloading. IEEE Internet Things J..

[34] Yang D.-Y., Chen Y.-H., Wu C.-H. (2020). Modelling and optimisation of a two-Server queue with multiple vacations and working breakdowns. Int. J. Prod. Res..

[35] Gu X., Ji C., Zhang G. (2020). Energy-Optimal Latency-Constrained Application Offloading in Mobile-Edge Computing. Sensors.

[36] Rodrigues T.G., Suto K., Nishiyama H., Kato N. (2017). Hybrid Method for Minimizing Service Delay in Edge Cloud Computing Through VM Migration and Transmission Power Control. IEEE Trans. Comput..

[37] Zhang J., Ren F., Shu R., Huang T., Liu Y. (2016). Guaranteeing Delay of Live Virtual Machine Migration by Determining and Provisioning Appropriate Bandwidth. IEEE Trans. Comput..

[38] Khazaei H., Miic J., Miic V.B., Mohammadi N.B. Modeling the Performance of Heterogeneous IaaS Cloud Centers. Proceedings of the 2013 IEEE 33rd International Conference on Distributed Computing Systems Workshops.

[39] Liu B., Chang X., Liu B., Chen Z. Performance Analysis Model for Fog Services under Multiple Resource Types. Proceedings of the 2017 International Conference on Dependable Systems and Their Applications (DSA).

[40] Fernando D., Terner J., Gopalan K., Yang P. Live Migration Ate My VM: Recovering a Virtual Machine after Failure of Post-Copy Live Migration. Proceedings of the IEEE INFOCOM 2019-IEEE Conference on Computer Communications.

[41] Groesbrink S. Virtual Machine Migration as a Fault Tolerance Technique for Embedded Real-Time Systems. Proceedings of the 2014 IEEE Eighth International Conference on Software Security and Reliability-Companion.

[42] Callegati F., Cerroni W. Live Migration of Virtualized Edge Networks: Analytical Modeling and Performance Evaluation. Proceedings of the 2013 IEEE SDN for Future Networks and Services (SDN4FNS).

[43] Chen P.-C., Lin C.-I., Huang S.-W., Chang J.-B., Shieh C.-K., Liang T.-Y. A Performance Study of Virtual Machine Migration vs. Thread Migration for Grid Systems. Proceedings of the 22nd International Conference on Advanced Information Networking and Applications-Workshops (Aina Workshops 2008).

[44] He T., Toosi N.A., Buyya R. (2019). Performance evaluation of live virtual machine migration in SDN-Enabled cloud data centers. J. Parallel Distrib. Comput..

[45] Kumar N., Zeadally S., Chilamkurti N., Vinel A. (2015). Performance analysis of Bayesian coalition game-Based energy-aware virtual machine migration in vehicular mobile cloud. IEEE Netw..

[46] Li S., Huang J. GSPN-Based Reliability-Aware Performance Evaluation of IoT Services. Proceedings of the 2017 IEEE International Conference on Services Computing (SCC).

[47] Liu H., Jin H., Xu C.-Z., Liao X. (2013). Performance and energy modeling for live migration of virtual machines. Cluster. Comput..

[48] Begam R., Wang W., Zhu D. (2020). TIMER-Cloud: Time-Sensitive VM Provisioning in Resource-Constrained Clouds. IEEE Trans. Cloud Comput..

[49] Chaufournier L., Sharma P., Le F., Nahum E., Shenoy P., Towsley D., Zhang J., Chiang M., Maggs B. (2017). Fast transparent virtual machine migration in distributed edge clouds. Proceedings of the Second ACM/IEEE Symposium on Edge Computing.

[50] Genez T.A.L., Tso F.P., Cui L. Latency-Aware joint virtual machine and policy consolidation for mobile edge computing. Proceedings of the 2018 15th IEEE Annual Consumer Communications & Networking Conference (CCNC).

[51] Wang X., Chen X., Yuen C., Wu W., Zhang M., Zhan C. (2017). Delay-Cost tradeoff for virtual machine migration in cloud data centers. J Netw. Comput. Appl..

[52] Elbamby M.S., Perfecto C., Liu C.-F., Park J., Samarakoon S., Chen X., Bennis M. (2019). Wireless Edge Computing With Latency and Reliability Guarantees. Proc. IEEE.

[53] Liu Y., Li R., Li Q. Reliability Analysis of Cloud Computing Systems with Different Scheduling Strategies under Dynamic Demands. Proceedings of the 2017 4th International Conference on Information Science and Control Engineering (ICISCE).

[54] Klimenok V., Breuer L., Tsarenkov G., Dudin A. (2005). The tandem queue with losses. Perform. Eval..

[55] Gómez-Corral A., Martos M.E. (2009). Marked Markovian Arrivals in a Tandem G-Network with Blocking. Methodol. Comput. Appl. Probab..

[56] Phung-Duc T. (2012). An explicit solution for a tandem queue with retrials and losses. Oper. Res. Int. J..

[57] Kim C., Dudin A., Dudina O., Dudin S. (2014). Tandem queueing system with infinite and finite intermediate buffers and generalized phase-type service time distribution. Eur. J. Oper. Res..

[58] Wu K., Shen Y., Zhao N. (2017). Analysis of tandem queues with finite buffer capacity. IISE Trans..

[59] Li R., Huang N., Kang R. Modeling and simulation for network transmission time reliability. Proceedings of the 2010 Proceedings-Annual Reliability and Maintainability Symposium (RAMS).

[60] He W., Liu X., Zheng L., Yang H. Reliability Calculus: A Theoretical Framework to Analyze Communication Reliability. Proceedings of the 2010 IEEE 30th International Conference on Distributed Computing Systems.

[61] Shen J., He W.-b., Liu X., Wang Z.-b., Wang Z., Yao J.-G. (2015). End-To-End delay analysis for networked systems. Front. Inf. Technol. Electron. Eng..

[62] Burke P. Output process and tandem queues. Proceedings of the Symposium on Computer-Communications Networks and Teletraffic.

[63] Gass S.I., Fu M.C. (2013). Encyclopedia of Operations Research and Management Science.

[64] Ross S.M. (2019). Queueing Theory. Introduction to Probability Models.

[65] Sarker V.K., Queralta J.P., Gia T.N., Tenhunen H., Westerlund T. A Survey on LoRa for IoT: Integrating Edge Computing. Proceedings of the 2019 Fourth International Conference on Fog and Mobile Edge Computing (FMEC).

[66] Ortin J., Cesana M., Redondi A. (2019). Augmenting LoRaWAN Performance with Listen Before Talk. IEEE Trans. Wirel. Commun..

